# MyD88 and STING Signaling Pathways Are Required for IRF3-Mediated IFN-β Induction in Response to *Brucella abortus* Infection

**DOI:** 10.1371/journal.pone.0023135

**Published:** 2011-08-02

**Authors:** Leonardo A. de Almeida, Natalia B. Carvalho, Fernanda S. Oliveira, Thais L. S. Lacerda, Anilton C. Vasconcelos, Lucas Nogueira, Andre Bafica, Aristóbolo M. Silva, Sergio C. Oliveira

**Affiliations:** 1 Department of Biochemistry and Immunology, Institute of Biological Sciences, Federal University of Minas Gerais, Belo Horizonte-Minas Gerais, Brazil; 2 Department of Pathology, Institute of Biological Sciences, Federal University of Minas Gerais, Belo Horizonte-Minas Gerais, Brazil; 3 Department of Morphology, Institute of Biological Sciences, Federal University of Minas Gerais, Belo Horizonte-Minas Gerais, Brazil; 4 Department of Microbiology, Immunology and Parasitology, Federal University of Santa Catarina, Florianopolis-Santa Catarina, Brazil; University of São Paulo, Brazil

## Abstract

Type I interferons (IFNs) are cytokines that orchestrate diverse immune responses to viral and bacterial infections. Although typically considered to be most important molecules in response to viruses, type I IFNs are also induced by most, if not all, bacterial pathogens. In this study, we addressed the role of type I IFN signaling during *Brucella abortus* infection, a facultative intracellular bacterial pathogen that causes abortion in domestic animals and undulant fever in humans. Herein, we have shown that *B. abortus* induced IFN-β in macrophages and splenocytes. Further, IFN-β induction by *Brucella* was mediated by IRF3 signaling pathway and activates IFN-stimulated genes via STAT1 phosphorylation. In addition, IFN-β expression induced by *Brucella* is independent of TLRs and TRIF signaling but MyD88-dependent, a pathway not yet described for Gram-negative bacteria. Furthermore, we have identified *Brucella* DNA as the major bacterial component to induce IFN-β and our study revealed that this molecule operates through a mechanism dependent on RNA polymerase III to be sensed probably by an unknown receptor via the adaptor molecule STING. Finally, we have demonstrated that IFN-αβR KO mice are more resistant to infection suggesting that type I IFN signaling is detrimental to host control of *Brucella*. This resistance phenotype is accompanied by increased IFN-γ and NO production by IFN-αβR KO spleen cells and reduced apoptosis.

## Introduction

*Brucella abortus* is a Gram-negative, facultative intracellular coccobacillus which causes brucellosis in humans and in cattle. In humans *B. abortus* causes undulant fever, endocarditis, arthritis and osteomyelitis and, in animals, it leads to abortion and infertility resulting in serious economic losses [1], [2]. The protective response against *B. abortus* infection requires CD4^+^ and CD8^+^ T lymphocytes, Th1-type cytokines such as interferon-gamma (IFN-γ) and tumor necrosis factor (TNF-α), and activated macrophages and dendritic cells [3]–[5]. The innate immune system is the first line of defense mechanisms that protect hosts from invading *Brucella*. It begins with the recognition of pathogen-associated molecular patterns (PAMPs) by pattern recognition receptors (PRRs). The best characterized PRRs known to recognize *Brucella* PAMPs are the Toll-like receptors (TLRs), which are transmembrane receptors that sense lipids, lipoproteins, proteins and nucleic acids [6]–[8]. This recognition activates intracellular signaling pathways that culminate in the induction of inflammatory cytokines, chemokines, interferons and upregulation of co-stimulatory molecules [9]. It has been shown that *Brucella* is recognized by several TLR-associated pathways triggering proinflammatory responses that impact both the nature and the intensity of the immune response [10].

Since their discovery nearly 50 years ago, IFNs have been described as products of virus-infected cells capable of inducing an antiviral state in neighbouring cells through a paracrine signaling [11]. Conversely, type I IFNs have been traditionally assigned a minor role in antibacterial host defenses [12]. Recently, however, conserved bacterial products, such as LPS and DNA, were found to activate distinctive signal transduction pathways compared to those activated by viruses and lead to high levels of type I IFN production [13]. Moreover, the role of type I IFN in antibacterial defenses is still controversial. The induction of type I IFN during some intracellular Gram-positive bacterial pathogens infection appears to be detrimental to the host. In IFN-α/β-receptor KO mice infected with *Listeria monocytogenes* the titer of bacteria recovered from the liver and spleen was 10^2^–10^3^-fold lower than in the wild type mice [14], [15]. Another intracellular bacterium that induces high levels of type I IFN is a hypervirulent strain of *Mycobacterium tuberculosis*. It was showed that mice infected with this *M. tuberculosis* strain died at faster rate due to failure to induce Th1 cellular mediated immunity [16]. The lack of development of Th1 immunity in response to this bacterium appears to be associated with increased induction of type I IFNs. In the *Chlamydia muridarum* infection model, IFN-α/β-receptor KO mice were found to be more resistant to infection than wild type mice [17]. The observed susceptibility of the wild type mice to *C. muridarum* was due to the induction of type I IFNs resulting in enhanced apoptosis of lung infiltrated macrophages. However, there are other bacterial infection models such as *Legionella pneumophila*, *Bacillus anthracis* and Group B *Streptococcus* where the function of type I IFNs are protective for host suppressing bacterial invasion due the enhancement of IFN-γ/NO production [18]–[20].

Although TLRs are the best characterized innate receptors related to recognize and to trigger a signaling for an efficient response against pathogens, they are only a piece of a broad repertoire of molecules capable to induce cellular signaling to fight against a microbial infection. Among them, the cytosolic receptors which are capable of sensing pathogen nucleic acids released from lysed bacteria or from bacterial secretion system during infection have been intensively studied [13]. Regarding that, retinoic acid inducible gene I (RIG-I), melanoma differentiation associated gene 5 (MDA5) and laboratory of genetics and physiology 2 (LGP2), members of RLR family were reported to recognize viral RNA and trigger an anti-viral response inducing type I IFN [21]. Few reports are available describing the role of RLR receptors recognizing bacterial RNAs. Since bacterial mRNAs are not capped and can contain 5′ triphosphate, they can induce type I interferon throughout RIG-I signaling [22]. In independent studies, Ablasser and colleagues [23], and Chiu et al. [24] demonstrated that RNA polymerase III transcribes highly AT-rich bacterial DNA present in the host cell cytosol to 5′-triphosphate RNA which is preferentially recognized by RIG-I [25]. The downstream signaling mediated by RIG-I requires the protein adaptor IPS-1 (MAVS, Cardiff or VISA), which coordinates the activation of IRF3/7, NF-κB and MAP kinases [21]. In addition to IPS-1, another molecule termed stimulator of interferon genes (STING) has been identified as an adaptor required by RIG-I to induce type I IFN in response to *Listeria monocytogenes* and *C. muridarum* [26], [27]. Furthermore, other groups have attempted to find a yet undefined bacterial DNA sensor that can signal through a TLR9-independent and RLR-independent pathways [28], [29].

In this study, we have shown that *B. abortus* induced IFN-β in macrophages and splenocytes. To gain insights into the molecules involved in IFN-β synthesis we defined that *Brucella* DNA is a potent stimulus for this cytokine expression. In addition, IFN-β induced by *Brucella* requires IRF3 and STAT1 phosphorylarion at tyrosine 701. Further, *Brucella* or its DNA induces IFN-β independent of TLRs and TRIF signaling but dependent on MyD88. Regarding, the putative cytosolic sensors involved in *Brucella* DNA recognition, we demonstrated here that RNA polymerase III and the adaptor molecule STING are critical components required for IFN-β signaling pathway. Additionally, we have demonstrated that IFN-αβR KO mice are more resistant to infection suggesting that type I IFN signaling is detrimental to host control of *Brucella*. This resistance phenotype seems to be related to increased IFN-γ and NO production by IFN-αβR KO spleen cells and reduced apoptotic profile. In summary, these findings propose a novel IFN-β pathway induced by a Gram-negative bacterial DNA that requires the engagement of RNA polymerase III and STING.

## Results

### *Brucella* induces IFN-β in macrophages and splenocytes

**Figure 1:**
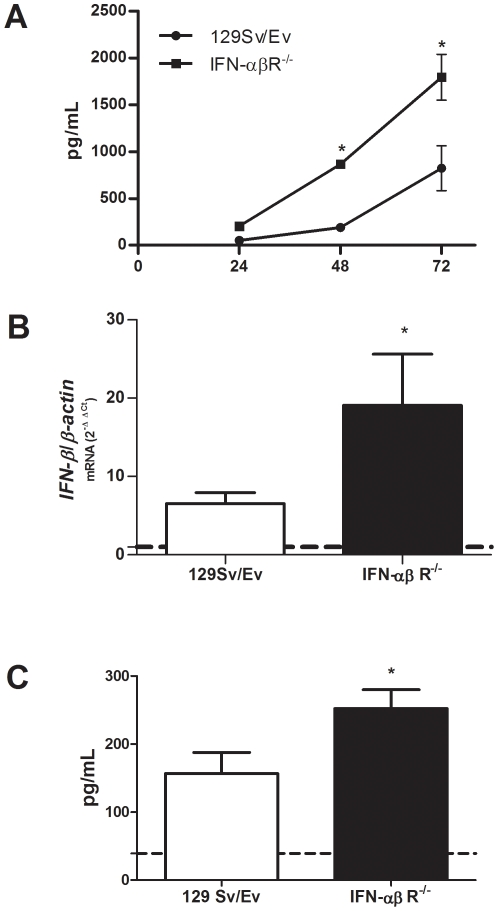
*Brucella* induces IFN-β in macrophages and spleen cells. (A) BMDM from 129Sv/Ev or IFN-αβR KO mice were infected with *B. abortus* strain S2308 (10^3^ bact/cell) and IFN-β was measured by ELISA at 24, 48 and 72 hours. (B) Spleen cells from *B. abortus* infected mice at fifteen days post-infection were isolated and total RNA was harvested and mRNA levels of *IFN-β* were determined by real-time RT-PCR and normalized to *β-actin*. (C) Spleen cells from *B. abortus* infected mice at fifteen days post-infection were cultured with 10^2^ bacteria/cell for 48 hrs and IFN-β was detected by ELISA. Error bars represent the mean ±SD. Similar results were obtained in two-independent experiments. Statistically significant difference of IFN-β levels from IFN-αβR KO mice compared to wild type is denoted by an asterisk (p<0.05).

To investigate whether *B. abortus* induces type I IFN, macrophages or spleen cells were exposed to bacteria and IFN-β was measured at several time points. Wild type (129Sv/Ev) or IFN-αβR KO macrophages as well as splenocytes infected with *B. abortus* strain 2308 displayed increased expression of IFN-β as measured by ELISA or real-time RT-PCR, respectively. In infected BMDM, maximal IFN-β responses were observed at 72 hrs post-infection (Fig. 1A). Curiously, IFN-αβR KO macrophages produced greater amounts of IFN-β compared to the wild type cells. This process is probably a compensatory mechanism due to the lack of the type I IFN receptor. Furthermore, fifteen days after infection *IFN-β* transcripts were measured in splenocytes by real-time RT-PCR (Fig. 1B) and IFN-β production by ELISA (Fig. 1C). Similarly to what was observed in macrophages, IFN-β was higher in IFN-αβR KO spleen cells compared to 129Sv/Ev mice. These findings demonstrate that *B. abortus* induces IFN-β production in immune cells.

### Absence of type I IFN signaling enhances host protection to *Brucella* infection

**Figure 2:**
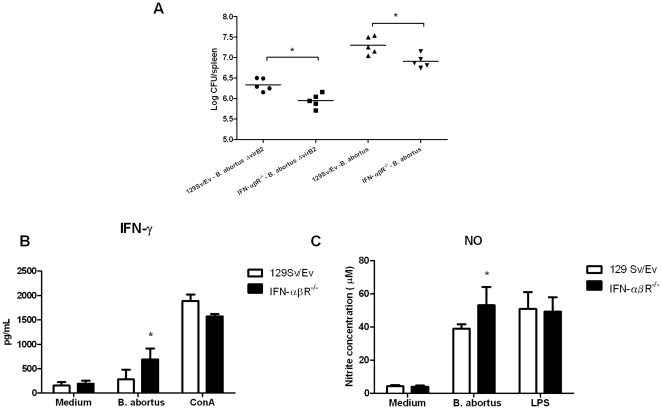
Bacterial burden, IFN-γ and NO production in *Brucella*-infected 129Sv/Ev and IFN-αβR KO mice. Five 129Sv/Ev or IFN-αβR KO mice were infected i.p. with a dose of 10^6^ CFU of *B. abortus* strain S2308 or Δ*virB* mutant. (A) Spleens were harvested at fifteen days post-infection, and the number of CFU in disrupted tissue was determined by 10-fold serial dilution and plating. (B) Spleen cells from *B. abortus* S2308 infected mice were cultured with 10^2^ bacteria/cell, ConA (5 µg/ml) or medium alone for 72 hrs and IFN-γ was detected by ELISA. (C) Regarding nitric oxide, nitrite concentration was measured by Griess reaction (µM) using the same stimuli described for IFN-γ measurements except the use of *E. coli* LPS (1 µg/ml) as positive control. Error bars represent the mean ±SD. Similar results were obtained in three-independent experiments. Statistically significant difference from IFN-αβR KO mice compared to wild type is denoted by an asterisk (p<0.05).

To study the role of type I IFN in host response against *Brucella* infection, we infected IFN-αβR KO and 129Sv/Ev mice with 1×10^6^ *B. abortus* strain 2308. Comparison of colony-forming units (cfu) recovered from the spleens of IFN-αβR KO and 129Sv/Ev mice fifteen days post-infection showed reduced bacterial numbers in type I IFN receptor deficient mice (Figure 2A). Similar findings demonstrating the detrimental role of type I IFN signaling to host control of bacterial infection were also observed for *L. monocytogenes*, *Tropheryma whipplei* and *C. muridarum* [14], [15], [17], [30]. Since *Brucella* mutant to the type IV secretion system (T4SS, *virB* mutant) fails to elicit IFN-induced gene expression during early infection [31], we decided to also evaluate the role of T4SS during *Brucella* infection in IFN-αβR KO mice. As observed in *Brucella* strain S2308 expressing T4SS infection, *virB* mutant showed reduced cfu counts in type I IFN receptor deficient mice compared to the wild type (Figure 2A). However, the reduced numbers of *virB* mutant cfu found in both mouse strains compared to parental strain 2308 is probably due to already described reduced virulence of this mutant [32]. These results demonstrated that T4SS plays no role in resistance of IFN-αβR KO mice to *B. abortus* infection in vivo.

To address whether increased resistance observed in IFN-αβR KO mice was associated with pro-inflammatory mediators, we measured IFN-γ, IL-17 and NO in spleen cells of type I receptor deficient mice and control animals. Elevated production of IFN-γ and NO in *Brucella*-primed splenocytes in vitro stimulated with live strain 2308 was observed in receptor-deficient mice compared to the wild type (Figure 2B and 2C). Additionally, no difference in IL-17 production was observed in IFN-αβR KO compared to 129Sv/Ev mice (Figure S1). Together, these findings suggest that type I IFN signaling induced by *Brucella* is detrimental to infection and regulate inflammatory components required for host protection.

### *Brucella abortus*-induced splenic apoptosis is dependent on type I IFN signaling

**Figure 3:**
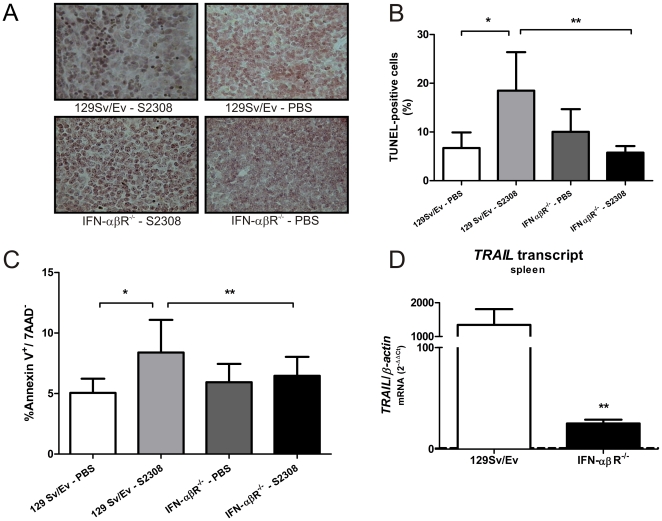
*B. abortus* induced-apoptosis depends on type I IFN signaling. (A) Spleens from 129Sv/Ev or IFN-αβR KO mice after two-weeks of infection with *B. abortus* were removed and tissue sections were analyzed to determine the apoptotic cells by TUNEL assay as described in material and methods. (B) This graph demonstrates the % of TUNEL-positive cells shown in panel A. (C) Annexin V^+^/7-AAD^−^ analysis of spleen cells of *Brucella*-infected 129Sv/Ev or IFN-αβR KO mice performed by flow cytometry. (D) The expression levels of *TRAIL* were measured by real-time PCR in spleens of *Brucella*-infected 129Sv/Ev or IFN-αβR KO mice. Similar results were obtained in two-independent experiments. Error bars represent the mean ±SD. Statistically significant difference from *Brucella*-infected and uninfected mice is denoted by an asterisk and between IFN-αβR KO mice compared to wild type is denoted by two asterisks (p<0.05).

One of the hallmark effects mediated by type I IFNs is the induction of apoptosis in infected cells which has been shown in the *L. monocytogenes* model to be associated with enhanced infection [14]. To assess whether *B. abortus* can also induce apoptosis, mice were infected and splenocyte apoptosis was determined by TUNEL assay and by flow cytometry using annexin V. We have observed increased apoptosis in the splenic tissue of 129Sv/Ev infected mice compared to uninfected controls as indicated by TUNEL+ cells (Figure 3A and 3B). However, *Brucella*-infected IFN-αβR KO mouse spleens exhibited a drastic reduction in splenic apoptosis compared to wild type controls. The results presented in Figure 3B as % of TUNEL-positive cells were determined by counting TUNEL+ cells in mouse spleen sections. These results were also confirmed by the percentage of Annexin V^+^/7AAD^−^ cells detected in spleens. IFN-αβR KO splenocytes infected with *Brucella* did not show enhancement in the number of Annexin V^+^/7-AAD^−^ cells compared to uninfected controls; however, it was observed in 129Sv/Ev mice (Figure 3C). In order to detect the type I IFN-stimulated genes (ISGs) up-regulated in spleens during *Brucella* infection, we measured the expression of *TRAIL*, *IP-10* and *IRF7* by real-time RT-PCR. Figure 3D shows that only the proapoptotic gene *TRAIL* was significantly up-regulated in *Brucella*-infected splenocytes from wild type mice and it was highly suppressed in IFN-αβR KO mouse spleens. For *IP-10* and *IRF7*, we did not observe statistically significant difference in gene expression in receptor-deficient mice compared to the wild type (data not shown). These results demonstrate that *B. abortus*-induced apoptosis in vivo is largely dependent on type I IFN signaling.

### Induction of *IFN-β* and *TRAIL* expression requires IRF3

**Figure 4:**
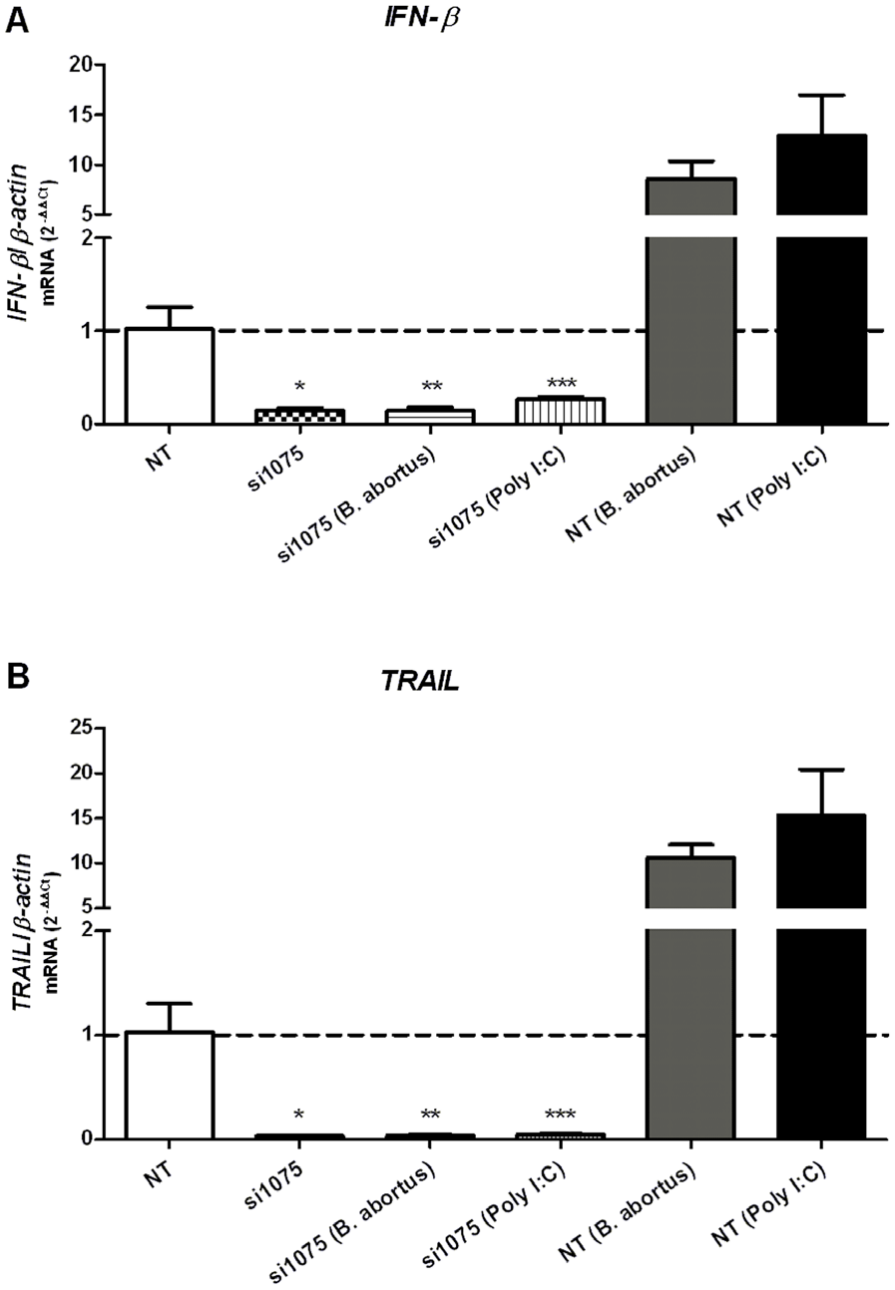
IRF3-dependent expression of IFN-β and TRAIL upon *Brucella abortus* infection. Raw 264.7 macrophages were treated with non-targeting control siRNA, a specific-targeting IRF3 (si1075) or non-treated control (NT). Total mRNA was obtained after 24 hrs stimulation with *B. abortus* S2308 (10^3^ bact/cell) or poly I∶C (10 µg/ml) as positive control. Next, *IFN-β* (A) and *TRAIL* (B) transcript levels were measured by real-time RT-PCR. Error bars represent the mean ±SD of samples assayed in triplicate. A representation of at least three experiments is shown. Statistically significant difference from cells treated with si1075 versus non-treated cells is denoted by an asterisk, from *Brucella*-infected cells treated with si1075 versus non-treated (NT) cells by two asterisks and from poly I∶C stimulated cells treated with si1075 versus non-treated cells by three asterisks (p<0.05).

The transcription factor IRF3 has been shown to mediate the induction of *IFN-β* gene in response to pathogens and TLR signaling [33]. To determine whether induction of IFN-β by *B. abortus* also involves IRF3, Raw 264.7 macrophages were treated with siRNA 1075 specific for IRF3 and the cells infected with *Brucella* S2308 or stimulated with poly I∶C as positive control. Transfection of IRF3-specific siRNA in Raw 264.7 cells resulted in a dramatic reduction of *IFN-β* expression levels (90%) as determined by real-time RT-PCR (Figure 4A). Inhibition of IRF3 led to a profound reduction of *IFN-β* transcripts following *B. abortus* or poly I∶C stimulation, compared to siRNA non-treated cells. This result identifies IRF3 as a component of the signaling pathway leading to type I IFN induction by *B. abortus*-infected macrophages. Similarly to what was observed for IFN-β, IRF3 inhibition resulted in a dramatic reduction in *TRAIL* expression (95%) in *Brucella*-infected macrophages (Figure 4B). Taken together, these findings demonstrated that IRF3 is a critical transcriptional regulator of *IFN-β* and *TRAIL* gene expression during *B. abortus* infection.

### *Brucella* DNA induces *IFN-β* and *TRAIL* expression in macrophages

**Figure 5:**
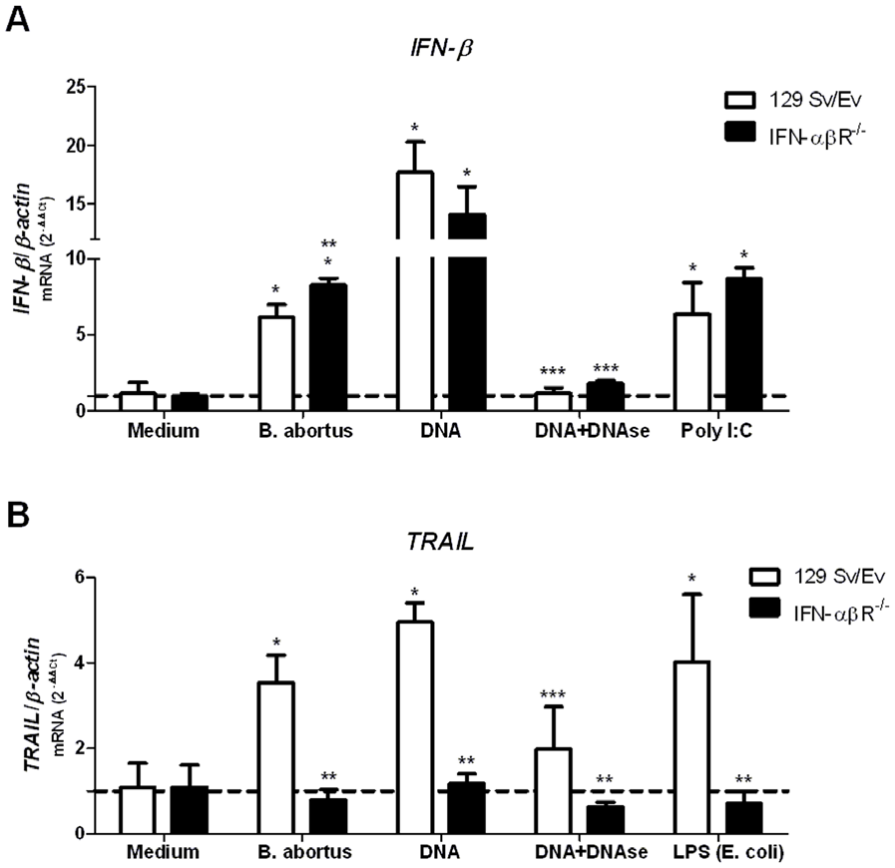
*Brucella* DNA is the major component responsible for IFN-β and TRAIL expression in macrophages. BMDM from 129Sv/Ev or IFN-αβR KO mice were infected with *B. abortus* strain S2308 (10^3^ bact/cell), stimulated with *Brucella* DNA (1 µg/5×10^5^ cells), *Brucella* DNA treated with DNAse (0.2 U/µg DNA), poly I∶C (10 µg/ml) or medium alone. After that, total RNA was harvested and mRNA levels of *IFN-β* (A) and *TRAIL* (B) were determined by real-time RT-PCR and normalized to *β-actin*. Error bars represent the mean ±SD of samples assayed in triplicate. Similar results were obtained in two-independent experiments. Statistically significant difference of *IFN-β* or *TRAIL* levels from stimulated macrophages versus non-stimulated cells is denoted by an asterisk, from IFN-αβR KO mouse macrophages compared to wild type is denoted by two asterisks and from *Brucella* DNA compared to *Brucella* DNA treated with DNAse with three asterisks (p<0.05).

Recent evidences suggest that induction of type I IFN by bacteria is mediated by nucleic acids from different pathogens [34]. In order to identify specific *Brucella* components involved in *IFN-β* expression, we have tested *Brucella* DNA, Omp19 (outer-membrane lipoprotein) or LPS. As observed in Figures 5A and 5B, *Brucella* DNA induces high levels of *IFN-β* and *TRAIL* expression in 129Sv/Ev BMDM. In contrast, reduced transcripts for *TRAIL* but not for *IFN-β* were observed in DNA-exposed macrophages from IFN-αβR KO mice. These results lead us to conclude that *IFN-β* expression induced by *Brucella* or its DNA occurs independently of type I IFN receptor signaling. *Brucella* Omp19 did not induce the expression of *IFN-β* and *Brucella* LPS induced very low levels of this cytokine mRNA (Figure S2). Treatment of *Brucella* DNA with DNase I abolished the stimulatory activity, a result suggesting that the DNA is the main ligand for an unknown receptor responsible for triggering the IFN-β response.

### STAT1 phosphorylation induced by *Brucella* or its DNA is dependent on type I IFN signaling

**Figure 6:**
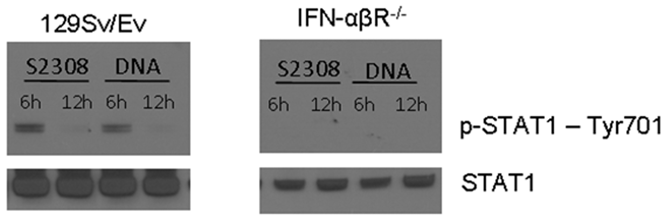
STAT1 phosphorylation induced by *B. abortus* or its DNA is impaired in mice lacking IFN-αβ receptor. BMDMs from wild type (129Sv/Ev) and IFN-αβR KO mice were stimulated for 6 and 12 hrs with *B. abortus* S2308 (10^3^ bact/cell) or *Brucella* DNA (1 µg/5×10^5^ cells). Cell lysates were analyzed by immunoblotting using anti-STAT1 and anti-phospho-STAT1 at tyrosine 701 residue. One experiment is shown representative of three.

Once produced type I IFNs can signal in an autocrine and paracrine manner leading to the phosphorylation-dependent activation of STAT1 through type I IFN receptor [35]. To determine whether *B. abortus* or its DNA induces STAT1 activation, we performed western blot analysis using specific antibodies targeting STAT1 phosphorylated at tyrosine 701 in BMDM. As shown in Figure 6, an increased STAT1 Tyr701 phosphorylation after *B. abortus* or its DNA stimulation was evidenced at 6 hrs but not at 12 hrs in 129Sv/Ev mice. In contrast, STAT1 phosphorylation was completely absent when IFN-αβR KO BMDM were infected with *Brucella* or activated with its DNA.

### *Brucella-i*nduced *IFN-β* expression is TLR- and TRIF-independent but MyD88-dependent

**Figure 7:**
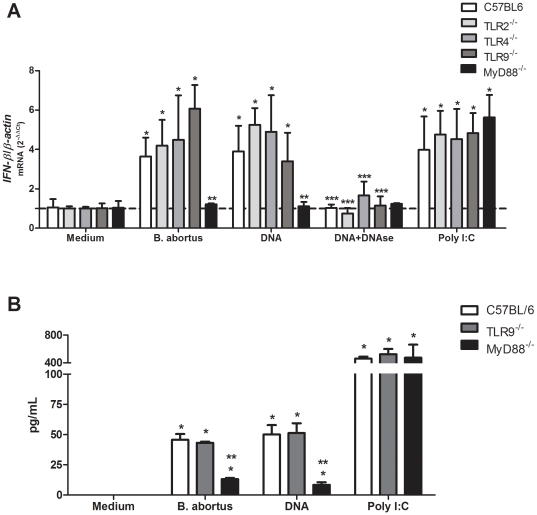
*Brucella*-induced IFN-β expression requires MyD88 signaling independently of TLR2, TLR4, and TLR9. (A) BMDM from TLR2, TLR4, TLR9 and MyD88 KO and wild type (C57BL/6) mice were infected with *B. abortus* strain S2308 (10^3^ bact/cell), stimulated with *Brucella* DNA (1 µg/5×10^5^ cells), *Brucella* DNA treated with DNAse (0.2 U/µg DNA), poly I∶C (10 µg/ml) or medium alone. After 24 hours, total RNA was harvested and mRNA levels of *IFN-β* were determined by real-time RT-PCR and normalized to *β-actin*. Statistically significant difference of *IFN-β* levels from stimulated macrophages versus non-stimulated cells is denoted by an asterisk, from MyD88KO compared to the other mice by two asterisks and from *Brucella* DNA compared to *Brucella* DNA treated with DNAse with three asterisks (p<0.05). (B) BMDM cells from TLR9 KO, MyD88 KO and wild type (C57BL/6) were infected with *B. abortus* strain S2308 (10^3^ bact/cell), stimulated with *Brucella* DNA (1 µg/5×10^5^ cells), poly I∶C (10 µg/ml) or medium alone were cultured for 24 hrs and IFN-β was detected by ELISA. Statistically significant difference of IFN-β levels from stimulated macrophages versus non-stimulated cells is denoted by an asterisk, from MyD88KO compared to the other mice by two asterisks. Error bars represent the mean ±SD of samples assayed in triplicate. Similar results were obtained in three-independent experiments.

**Figure 8:**
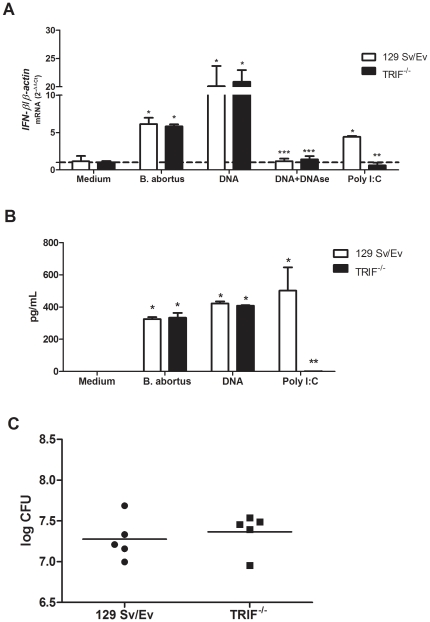
TRIF-mediated signaling is dispensable for IFN-β expression induced by *B. abortus* or its DNA. (A) BMDMs from TRIF KO and wild type (129Sv/Ev) mice were infected with *B. abortus* strain S2308 (10^3^ bact/cell), stimulated with *Brucella* DNA (1 µg/5×10^5^ cells), *Brucella* DNA treated with DNAse (0.2 U/µg DNA), poly I∶C (10 µg/ml) or medium alone. After 24 hours, total RNA was harvested and mRNA levels of *IFN-β* were determined by real-time PCR and normalized to *β-actin*. Error bars represent the mean ±SD of samples assayed in triplicate. Similar results were obtained in three-independent experiments. Statistically significant difference of *IFN-β* levels from stimulated macrophages versus non-stimulated cells is denoted by an asterisk, from wild type mice to TRIF KO with two asterisks and from *Brucella* DNA compared to *Brucella* DNA treated with DNAse with three asterisks (p<0.05). (B) BMDM cells from TRIF KO and wild type (129Sv/Ev) were infected with *B. abortus* strain S2308 (10^3^ bact/cell), stimulated with *Brucella* DNA (1 µg/5×10^5^ cells), poly I∶C (10 µg/ml) or medium alone were cultured for 24 hrs and IFN-β was detected by ELISA. Statistically significant difference of IFN-β levels from stimulated macrophages versus non-stimulated cells is denoted by an asterisk, from TRIF KO compared to the wild type mice by two asterisks. Error bars represent the mean ±SD of samples assayed in triplicate. (C) Five 129Sv/Ev and TRIF KO mice were infected i.p. with a dose of 10^6^ CFU of *B. abortus* strain S2308. Spleens were harvested at fifteen days post-infection, and the number of CFU in disrupted tissue was determined by 10-fold serial dilution and plating. Similar results were obtained in three-independent experiments.

To determine the role of TLRs, TRIF and MyD88 signaling in IFN-β expression elicited by *Brucella* or its DNA, real-time RT-PCR was performed in TLR2, TLR4, TLR9, TRIF and MyD88 KO BMDM. As shown in Figure 7A and Figure 8A, IFN-β expression by *B. abortus* strain 2308 or its DNA is independent of TLR recognition and the adaptor molecule TRIF but dependent on MyD88 signaling. Our results demonstrated that in contrast to BMDM from wild type, TLRs KO or TRIF KO, *IFN-β* expression was totally abrogated in MyD88 BMDM infected with *Brucella* or stimulated with its DNA. To confirm these results we performed ELISA to measure IFN-β and we obtained similar results as observed for real-time PCR (Figure 7B and 8B). These findings suggest that other receptor signaling through MyD88 different from TLR2, TLR4 or TLR9 is involved in type I IFN response during *B. abortus* infection. Since the role of TRIF signaling during *B. abortus* infection in vivo has not been reported, we decided to determine the function of this adaptor molecule in host control of this bacterial infection. As shown in Figure 8C, fifteen days following infection with 1×10^6^ cfu of *B. abortus* strain 2308, bacterial numbers were similar in spleens of wild type and TRIF KO mice. Therefore, these results suggest that TRIF is not involved in the host control of *B. abortus* infection.

### *Brucella*-induced *IFN-β* is dependent on RNA Polymerase III and STING

**Figure 9:**
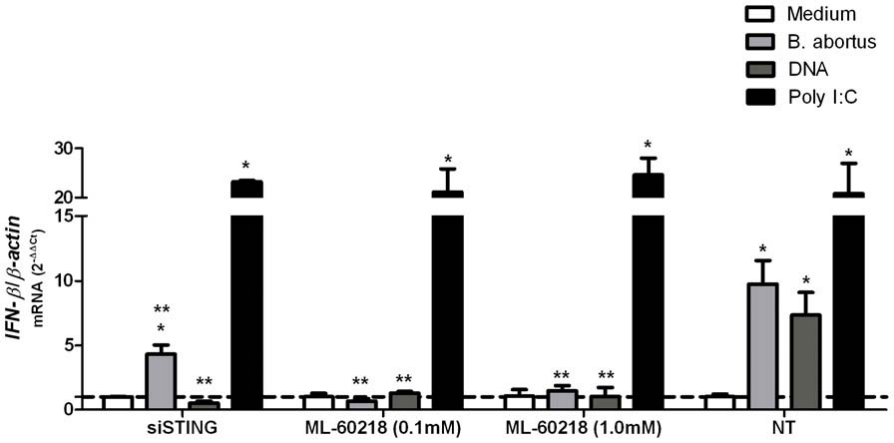
IFN-β expression induced by *B. abortus* or its DNA requires RNA polymerase III and the adaptor molecule STING. Raw 264.7 macrophages were treated with ML-60218 (RNA polymerase III inhibitor) for 10 hours and stimulated with *B. abortus* strain S2308 (10^3^ bact/cell), with *Brucella* DNA (1 µg/5×10^5^ cells), *Brucella* DNA treated with DNAse (0.2 U/µg DNA), poly I∶C (10 µg/ml) and medium alone. For STING knockdown, macrophages were treated with non-targeting control siRNA, a specif-targeting STING or non-treated control. After three hours of stimulation total RNA was harvested and mRNA levels of *IFN-β* were determined by real-time RT-PCR and normalized to *β-actin*. Error bars represent the mean ±SD of samples assayed in duplicate. Similar results were obtained in three-independent experiments. Statistically significant difference of *IFN-β* levels from *Brucella*- or its DNA-stimulated cells compared to unstimulated cells is denoted by an asterisk and from non-treated (NT) macrophages compared to siSTING or ML-60218 treated cells is denoted by two asterisks (p<0.05).

In addition to surface- or endosome-localized innate receptors, host cells also express several cytosolic sensors that induce type I IFN in response to nucleic acid ligands such as RNA and DNA [13]. However, the mechanisms by which bacteria stimulate cytosolic sensors are under investigation. Recently, Chiu et al. [24] reported that DNA could stimulate a RNA sensor. In this mechanism, RNA polymerase III transcribes cytosolic DNA thereby generating RNA ligands for RIG-I like receptors (RLR). In addition to the RNA polymerase III pathway, mouse and human cells have other cytosolic DNA sensors driving the transcriptional activation of type I IFN [29]. Further, STING (stimulator of interferon genes) was recently identified as a downstream signaling adaptor required for IFN-β induction in response to cytosolic DNA [26]. In order to address whether *B. abortus-* or bacterial DNA-induced *IFN-β* expression in Raw 264.7 macrophages was dependent on RNA polymerase III or STING, a pharmacological inhibitor of RNA polymerase III or siRNA technology for STING was used. As demonstrated in Figure 9, the use of RNA polymerase III inhibitior (ML-60218) at 0.1 or 1.0 mM of concentration dramatically reduced *IFN-β* mRNA during *Brucella* infection of macrophages (83%) or during stimulation with its DNA (100%). This finding demonstrates that RNA polymerase III-dependent pathway plays an important role in IFN-β induction by *Brucella*, probably transcribing bacterial DNA to be recognized by a yet unknown receptor. Furthermore, we tested the role of STING in *Brucella*-induced *IFN-β* expression. By real-time RT-PCR analysis, it can be observed that by silencing STING resulted in a significant decrease in *IFN-β* mRNA levels during *B. abortus* infection of macrophages (55%) or upon macrophage stimulation with bacterial DNA (93%) compared to siRNA non-treated cells. This result demonstrates that STING is a crucial adaptor molecule of host cell involved in IFN-β induction by *Brucella*.

## Discussion

Although type I IFNs are well known to induce a robust antiviral host response, the role of type I IFNs in response to bacterial infection is variable and even it is sometimes detrimental to the host. For instance, IFN-αβR KO mice exhibit lower *L. monocytogenes* burdens in the liver and spleen as compared with wild type mice [14], [15], [36]. Type I IFN signaling is also detrimental to the clearance of *M. tuberculosis* infection from the spleen [37] and the lung [38]. Furthermore, type I IFN impairs clearance of *Chlamydia* from the genital tract and lungs [17], [39], and it is detrimental to the host during infection with *T. whipplei* [30]. In contrast, type I IFN is crucial for host resistance to some bacterial infections. For example, IFN-αβR KO mice exhibit decreased survival and increased bacterial burdens upon infection with Group B *Streptococcus*, *Streptococcus pneumoniae* and *Escherichia coli* [20]. Additionally, type I IFN also plays a role in restricting *L. pneumophila* replication in macrophages [40]. The susceptibility of IFN-αβR KO mice to these infections has been correlated with reduced TNF-α and IFN-γ production. In response to *Francisella tularensis*, type I IFN signaling has also been observed to induce the expression of inflammasome components such as IL-1β and IL-18 [41]. Due to the complex cytokine circuit involving type I IFNs, the mechanisms by which the immunomodulatory effects of these IFNs are regulated are only beginning to be understood [42].

Regarding type I IFNs induced by *B. abortus* infection, the first demonstration that *Brucella* induces IFN-α was reported by Huang et al [8] using heat-killed bacteria (HKBa). IFN-α was detected in serum of wild type mice injected with HKBa and the level was markedly reduced in the TLR9 KO mice serum, demonstrating that HKBa induces IFN-α in a TLR9-dependent manner. Additionally, Salcedo et al [43] have shown that *Brucella* is able to induce IFN-β in dendritic cells. In this study, we have demonstrated that *Brucella* induces IFN-β in BMDM and splenocytes from wild type and IFN-αβR KO mice. Furthermore, we demonstrated that type I IFN signaling is detrimental to host control of *B. abortus* infection since low numbers of bacterial cfu were found in IFN-αβR KO mice when compared to the wild type control animals. In contrast to our findings, Roux et al [31] have shown that *Brucella* cfu recovered from IFN-αβR KO mice compared to parental BALB/c mice showed no significant differences during the first 4 weeks of infection. The differences observed in these studies may be related to the mouse strain used, since we have worked with 129Sv/Ev background and Roux et al [31] have used BALB/c mice. Further, the virB T4SS plays a crucial role in *Brucella* intracellular replication [32]. Since the virB T4SS system secretes effector proteins able to modify host cell functions, we also investigated whether virB was involved in *Brucella* host control in IFN-αβR KO mice. As demonstrated in Figure 2, similarly to observed to virulent strain 2308, IFN-αβR KO mice infected with *Brucella* Δ*virB* mutant showed reduced bacterial burden compared to wild type mice.

The in vivo mechanisms by which type I IFN signaling increases host susceptibility to bacterial infection remain unclear. One possible mechanism is that induction of type I IFN by *Listeria* was shown to suppress macrophage activation by reducing the ability to respond to IFN-γ down-regulating IFN-γR expression [44]. Mice lacking IFN-αβR have increased expression of IFN-γR and their reduced susceptibility to *L. monocytogenes* infection is dependent on IFN-γ. Herein, we observed that splenocytes from *Brucella*-infected IFN-αβR KO mice produced higher amounts of IFN-γ and NO, two critical components to host control of this bacterial infection [45]. Therefore, this mechanism of antagonistic cross-talk between type I and II IFNs might be an important aspect involved in IFN-αβR KO mice resistance to *Brucella* infection. Additionally, abundant type I IFN predisposes lymphocytes to apoptosis during *Listeria* infection and it has a negative effect on bacterial handling in the mouse [46]. In this study, we have observed that *Brucella*-infected 129Sv/Ev mice induced higher percentage of apoptotic spleen cells as demonstrated by TUNEL assay and by flow cytometry using annexinV^+^/7-AAD^−^. Conversely, mice lacking IFN-αβR were more resistant to *Brucella* infection and displayed less apoptotic lesions as well as reduced expression of pro-apoptotic gene *TRAIL* than their wild type counterparts. We speculate here that type I IFN signaling enhances immune cells apoptosis; therefore, causing the increased susceptibility to *Brucella*. However, further experiments are required to support this hypothesis. Another possible mechanism by which type I IFN enhances host susceptibility to bacterial infection, relates to the observation that these cytokines stimulate the production of IL-27, a molecule that strongly suppresses IL-17A production [47]. IL-17A produced by γδ T cells appears to play an important role in restricting *Listeria* replication by orchestrating neutrophil responses [48]. In fact, IFN-αβR KO mice induced more IL-17A in response to *Francisella* and *Listeria* infections [49]. We have investigated here whether *Brucella* regulates IL-17A production and we found that in bacteria-infected splenocytes the levels of IL-17A in IFN-αβR KO mice were similar to wild type spleen cells (Figure S1). This result ruled out the possibility that lack of type I IFN signaling could enhance IL-17A production and host resistance to *Brucella* infection.

Following stimulation with bacterial components, the constitutively expressed IRF3 is phosphorylated in the cytoplasm, dimerizes and then translocates into the nucleus to induce the transcription of type I IFN [50]. We therefore, determined the role of IRF3 in *IFN-β* and *TRAIL* expression in Raw 264.7 macrophages using siRNA technology. *IFN-β* and *TRAIL* expression were dramatically reduced when IRF3 transcription factor was silenced, identifying IRF3 as an important component of the signaling pathway leading to type I IFN induction by *Brucella*-infected macrophages. Next, we reasoned whether *B. abortus* induces IFN-inducible genes via a type I IFN autocrine loop after engagement of the type I receptor. Thus, we monitored STAT1 activation, one outcome of IFN secretion and type I IFN receptor engagement. *Brucella*-infected wild type BMDM showed an increased in STAT1 tyrosine 701 at 6 hrs post-infection. In contrast, STAT1 tyrosine 701 phosphorylation was totally abrogated in IFN-αβR KO macrophages infected with *Brucella*. Collectively, these findings demonstrate that *B. abortus* induction of IFN-β occurs through an IRF3-dependent pathway and that subsequent STAT1 activation via IFN-αβR leads to a positive feedback loop involved in BMDM response to *Brucella*.

Furthermore, we have compared the *IFN-β* expression induced by *Brucella* DNA, the lipoprotein Omp19 and its LPS. Our data supports that *Brucella* DNA is a major bacterial component to induce IFN-β in BMDM. Regarding TLR recognition of *Brucella*, we and others have shown previously that TLR2, TLR4 and TLR9 recognize bacterial components and induce pro-inflammatory cytokine production [6]–[8]. More recently, we have shown that the MyD88 adaptor molecule is crucial to host control against *Brucella* infection [9]. In this study, we aimed to examine the role of TLRs and their contribution to the *Brucella*-induced IFN-β response. Overall, the fact that expression of *IFN-β* was unimpaired in TLR2, TLR4 and TLR9 KO BMDM during infection strongly indicated that *Brucella*- or its DNA-induced *IFN-β* expression occurs mostly independent of TLRs. Surprisingly, type I IFN expression by *Brucella* or its DNA was dependent on MyD88 in BMDM. A pathway involving MyD88 and an unknown PRR recognizing various Gram-positive bacteria such as *Streptococcus* and *Bacillus anthracis* has been previously reported [51], [52]. However, to the best of our knowledge, activation of a MyD88-dependent, TLR2/TLR4/TLR9-independent signaling pathway is quite unique in Gram-negative bacteria. Recently, Lapaque et al [53] have reported that *Brucella*-induced IRGs expression (Irga6 and Irgm3) requires type I IFN production. Similarly to our findings, these authors revealed that Irga6 and Irgm3 induction by *B. abortus* is MyD88-dependent but TLR2/TLR4/TLR5/TLR9-independent. Since the adaptor molecule TRIF is involved in type I IFN production, we also decided to investigate whether *Brucella* could induce *IFN-β* expression through TRIF [54]. As shown in Figure 8A, *Brucella* or its DNA induces *IFN-β* expression in a TRIF-independent manner. Additionally, we addressed whether the adaptor molecule TRIF was involved in *Brucella* infection in vivo. Our results demonstrated that TRIF played no role in host control of *B. abortus*.

*Brucella* enters the host cell, prevents fusion of the phagosome with the lysosome by altering the intracellular traffic of the early phagosome vesicle [55] being located in structures that resemble the endoplasmic reticulum [56]. Therefore, DNA from dead *Brucella* is available in this endoplasmic reticulum-like organelle and/or escape to the cytosol compartment being available to bind to a cytosolic DNA sensor. Additionally, *Brucella abortus* expresses the virB T4SS that plays a crucial role in the bacterial replication [32]. Bacteria use type IV secretion systems for genetic exchange and to deliver effector molecules to eukaryotic target cells. In addition to DNA from dead *Brucella* that can be released in the BCV (*Brucella*-containing vacuole) and/or escape to the cytosol compartment being available to bind to a cytosolic DNA sensor, there is the possibility of the *Brucella* DNA be released by the translocation through the type IV secretion system. Even though, the virB was not involved in host control of *Brucella* in IFN-αβR KO mice, the role of the type IV secretion system in releasing bacterial DNA in the cytosol remains to be fully characterized during *B. abortus* infection. Furthermore, the cellular sensors that detect DNA and triggers type I IFN production have remained largely unknown. The cytosolic receptors which are capable of sensing pathogen nucleic acids released from lysed bacteria or from bacterial secretion system during infection are now under intensive investigation [13]. Regarding that, retinoic acid inducible gene I (RIG-I), melanoma differentiation associated gene 5 (MDA5) and laboratory of genetics and physiology 2 (LGP2), members of RLR family were reported to recognize viral RNA and trigger an anti-viral response inducing type I IFN [21]. In this study, our findings are consistent with the hypothesis that RNA polymerase III detects some segments of *Brucella* DNA and transcribes them into RNA ligands that induce IFN-β possibly through the RIG-I pathway. Since, inhibition of RNA polymerase III enzymatic activity totally abrogated *IFN-β* expression induced by *Brucella* DNA. These results strongly suggest that RNA polymerase III plays a key role in sensing bacterial DNA during *B. abortus* infection. Furthermore, an endoplasmic reticulum resident transmembrane protein termed STING (stimulator of interferon genes) has been identified as an adaptor required by RIG-I to induce type I IFN in response to *L. monocytogenes* and *C. muridarum* [26], [27]. In order to determine whether STING plays any role in *Brucella* sensing, we have used siRNA technology. siRNA silencing demonstrated that STING is also an important mediator of IFN-β induced by *Brucella* or its DNA functioning probably downstream of the RIG-I pathway or of an unknown receptor. Since STING was found to basally reside in the endoplasmic reticulum, similarly to *Brucella*, this cell compartment would be an important site that facilitates STING to signal during this bacterial infection. A recent study demonstrated that the IFN-β response to *L. monocytogenes* was lost in STING KO mouse embryonic fibroblasts [26], and in mouse oviduct epithelial cells and human cells during *C. muridarum* infection when siRNA was used [27]. These findings reinforce STING as an important component of the TLR-independent interferon response during bacterial infection.

In summary, our study demonstrated that *Brucella* induces IFN-β through IRF3 signaling pathway and activates IFN-stimulated genes via STAT1 phosphorylation. Additionally, *IFN-β* expression induced by *Brucella* is independent of TLRs but MyD88-dependent, pathway not yet described for Gram-negative bacteria. Further, being *Brucella* DNA a major bacterial component to induce IFN-β, our study suggests that this molecule is transcribed by RNA polymerase III to be sensed by an unknown receptor that signals via STING. To the best of our knowledge, this is the first report demonstrating that bacterial DNA can be sensed by RNA polymerase III/STING pathway to coordinate type I IFN production. Finally, type I IFN signaling enhances host susceptibility to *B. abortus* infection independently of bacterial T4SS. The mechanism involved in IFN-αβR KO mice resistance is unknown but it seems to involves enhanced IFN-γ and NO production and reduced apoptosis.

## Materials and Methods

### Ethics Statement

This study was carried out in strict accordance with the Brazilian laws 6638 and 9605 in Animal Experimentation. The protocol was approved by the Committee on the Ethics of Animal Experiments of the Federal University of Minas Gerais (Permit Number: CETEA89/2008).

### Mice, cell culture and bacteria

IFN-αβR KO mice were gifted by Dr. Luiz F. Reis (Ludwig Institute, São Paulo, Brazil). TLR2, TLR4, TLR9 or TRIF KO mice were kindly provided by Shizuo Akira from Osaka University in Japan. The wild-type strains 129Sv/Ev or C57BL/6 mice were purchased from the Federal University of Minas Gerais animal facility (UFMG, Belo Horizonte, Brazil). Genetically deficient and control mice were maintained at UFMG and used at 6–8 week of age. Mouse RAW 264.7 macrophages (American Type Culture Collection, ATCC Nu TIB-71) were grown in Dulbecco's Modified Eagle Medium (DMEM) high glucose containing 10% FCS and 1% penicillin G sodium (100 U/ml), and streptomycin sulfate (100 µg/ml). Bone marrow cells were obtained from femora and tibia of mice and they were derived in macrophages (BMDMs) as previously described [57]. *B. abortus* virulent strain 2308 was obtained from our own laboratory collection. The *virB* mutant strain was kindly provided by Dr. Renato de Lima Santos (UFMG, Belo Horizonte, Brazil). They were grown in *Brucella* broth medium (BD-Pharmingen, San Diego, CA) for 3 days at 37°C.

### Purification of genomic DNA from bacterial culture

To purify *Brucella* genomic DNA, a modification of the method described by Wilson [58] was used. Briefly, cells were resuspended in 0.5 ml of TE buffer (10 mM Tris-HCl, 1 mM EDTA, pH 8.0), heat killed at 80°C for 15 min, and incubated at 37°C for 1 h with 0.5% sodium dodecyl sulfate and proteinase K (200 mg/ml). Cell wall debris, denatured proteins, and polysaccharides were removed by precipitation of the lysate with 5 M NaCl and CTAB-NaCl solutions at 65°C for 10 min. DNA was extracted by a standard protocol with phenol-chloroform-isoamyl alcohol, precipitated with isopropanol, washed with 70% ethanol, and dried. The DNA-containing pellet was reconstituted in 100 µl of nuclease-free water containing RNAse (50 µg/mL). The concentration and purity of the DNA were determined spectrophotometrically.

### Infection and *Brucella* counts in spleens

Mice were infected i.p. with 10^6^ CFU of *B. abortus* strain 2308. To determine residual *Brucella* CFU in the spleens of mice, five animals from each group were examined at 2 weeks after infection. Spleens from individual animals were homogenized in PBS, 10-fold serially diluted, and plated on *Brucella* broth agar (Difco, BD-Pharmingen, San Diego, CA). Plates were incubated at 37°C and the number of CFU was counted after 3 days as previously described [57].

### IFN-γ, IFN-β, IL-17 and NO production by splenocytes

Splenocyte cultures from 129Sv/Ev or IFN-αβR KO mice were stimulated by addition of 10^2^ live *B. abortus* strain S2308 per cell or 1 µg/ml *Escherichia coli* LPS in a total volume of 200 µl of medium/well. Unstimulated cells were used as a negative control, and cells stimulated with Con A (5 µg/ml; Sigma-Aldrich, St. Louis, MO) were used as a T cell-activating control. Spleen cells were incubated at 37°C with 5% CO_2_. Levels of IFN-γ or IL-17 in the supernatants were measured using a commercially available ELISA Duoset kit (R&D Systems, Minnesota, MN). IFN-β levels were measured by Mouse Interferon-β ELISA Kit (PBL Biomedical Laboratories, Piscataway, NJ). To assess the amount of NO produced, spleen cell culture supernatants from IFN-αβR KO or 129 Sv/Ev mice stimulated with the same antigens described above were assayed for accumulation of the stable final product of NO, NO_2_ which was determined by the Griess reaction. Briefly, culture supernatants (50 µl) were mixed with 50 µl of Griess reagent (1% sulfanilamide, 0.1% naphthylethyline diamine dihydrochloride and 2.5% phosphoric acid) in triplicate in 96-well plates at room temperature for 5 min. The OD at 550 nm was then measured. NO_2_ concentration was determined by comparison with NaNO_2_ (Sigma, St. Louis, MO) as a standard.

### IFN-β production by BMDM and immunoblotting

BMDM cells from 129Sv/Ev, IFN-αβR or TRIF KO and C57BL/6, MyD88 or TLR9 KO animals completely differentiated into macrophages on day 10 of culture were stimulated with 10^3^ *B. abortus* strain S2308 bacteria per macrophage. The levels of IFN-β in the supernatants of 24 hrs stimulated BMDMs were measured by Mouse Interferon Beta ELISA Kit (PBL Biomedical Laboratories, Piscataway, NJ). For immunoblotting analysis, BMDM cells from IFN-αβR KO or 129 Sv/Ev mice were stimulated either with live *B. abortus* at 10^3^ bacteria/cell or 1 µg of purified *Brucella* DNA (1 µg/5×10^5^ cells). After 6 or 12 hours, cells were lysed with M-PER extraction buffer (Pierce, Rockford, IL) containing a phosphatase inhibitor mixture (Calbiochem, Darmstadt, Germany). Protein extracts (30 µg) were then separated by SDS-PAGE/NuPage system (Invitrogen Life Technologies, Carlsbad, CA), electroblotted onto a nitrocellulose membrane, and immunoblotted with specific antibodies for phospho-STAT1 (Y701) (1∶1000; Cell Signaling, Boston, MA) or for total STAT1 (1∶1000; Cell Signaling, Boston, MA). Antibody binding was detected with HRP-conjugated anti-IgG (1∶2000; Cell Signaling, Boston, MA), followed by ECL detection (GE Healthcare, Buckinghamshire, UK).

### Real-Time RT-PCR

Splenocytes from *Brucella* infected and non-infected 129Sv/Ev and IFN-αβR KO mice or BMDM cells from 129Sv/Ev, IFN-αβR KO, C57BL/6, TLR2 KO, TLR4 KO, TLR9 KO, MyD88 KO and TRIF KO mice were snap-frozen and stored in −80°C freezer until RNA isolation. Cells were homogenized in 350 µL of RA1 buffer and 3.5 µL of β-mercaptoethanol (illustra™ RNAspin Mini RNA Isolation Kit – GE Healthcare, Buckinghamshire, UK) and total RNA was isolated according to the manufacter's instruction. Reverse transcription of 1 µg from total RNA was performed using illustra™ Ready-To-Go RT-PCR Beads (GE Healthcare, Buckinghamshire, UK). Real-Time RT-PCR was conducted in a final volume of 10 µL containing the following: SYBR® Green PCR Master Mix (Applied Biosystems, Foster City, CA), oligo-dT cDNA as the PCR template and 20 µM of primers. The PCR reaction was performed with ABI 7900 Real-Time PCR System (Applied Biosystems, Foster City, CA), using the following cycling parameters: 60°C for 10 min, 95°C for 10 min, 40 cycles of 95°C for 15 sec and 60°C for 1 min, and a dissociation stage of 95°C for 15 sec, 60°C for 1 min, 95°C for 15 sec, 60°C for 15 sec. Primers were used to amplify a specific 100–120-bp fragment corresponding to specific gene targets as follows: *IFN-β* F: 5′-AGCTCCAAGAAAGGACGAACAT-3′, *IFN-β* R: 5′-GCCCTGTAGGTGAGGTTGATCT-3′, *TRAIL* F: 5′-ACCTCAGCTTCAGTCAGCACTTC-3′, *TRAIL* R: 5′-TGTAAGTCACAGCCACAGACACAG-3′, β-*Actin* F: 5′-AGGTGTGCACCTTTTATTGGTCTCAA-3′, β-*Actin* R: 5′-TGTATGAAGGTTTGGTCTCCCT-3′; *IRF3* F: 5′-GGACTTGCACATCTCCAACA-3′, *IRF3* R: 5′-TTGCTCCACGTAGGGACAAT-3′, *STING* F: 5′-CCTAGCCTCGCACGAACTTG-3′, *STING* R: 5′-CGCACAGCCTTCCAGTAGC-3′, *IP10* F: 5′-CCTGCCCACGTGTTGAGAT-3′, *IP10* R: 5′-TGATGGTCTTAGATTCCGGATTC-3′, *IRF7* F: 5′-CGCGGCACTAACGACAGGCGAG-3′, *IRF7* R: 5′-GCTGCCGTGCCCGGAATTCCAC-3′. All data is presented as relative expression units after normalization to the *β-actin* gene. PCR measurements were conducted in triplicate.

### TdT-mediated dUTP Nick-End Labeling (TUNEL) and Annexin V assays

Splenic tissue from infected and non-infected IFN-αβR KO or 129 Sv/Ev mice were removed and fixed in 4% paraformaldehyde. Four µm sections were cut and mounted onto poly-l-lysine–coated slides. The samples were permeabilized using 0.1% Triton and 0.1% sodium citrate, and then double stranded DNA breaks were labeled using an in situ FragEL™ DNA Fragmentation Detection Kit, Colorimetric - TdT Enzyme (Calbiochem, Darmstadt, Germany) according to the manufacturer's protocol. KS300 software contained in Carl Zeiss image analyzer was used for image acquisition of 50 images randomly chosen from each lamina at a microscope with 40× objective lens and scanned through microcamera JVC TK-1270/RGB. Then, the percentage of TUNEL positive cells was determined. For annexin V^+^ staining, splenocytes from *Brucella* infected and non-infected IFN-αβR KO or 129 Sv/Ev mice were obtained 2 weeks after infection and they were washed twice with cold PBS and then ressuspended in 1× Binding Buffer (BD-Pharmingen, San Diego, CA). Cells were stained with PE-annexin V^+^/7-AAD^−^ as recommended by the manufacturer's protocol (BD-Pharmingen, San Diego, CA). A minimum of 10,000 splenocyte-gated events were acquired using a FACScan apparatus (BD-Pharmingen, San Diego, CA). FACS results were analyzed using the FlowJo Software (Tree Star, Ashland, OR).

### siRNA and RNA Polymerase III inhibition

Primer sequences for silencing mouse IRF3 (accession number BC.050882.1) were predicted with siRNA Target Finder and siRNA Converter (Ambion® - Applied Biosystems, Foster City, CA) and further prepared with the Ambion Silencer™ siRNA kit. The primer sequences used were: AS-mIRF3-1075: 5′-AAGGTTGTTCCTACATGTCTTCCTGTC-3′, SENSE-mIRF3-1075 5′-AAAAGACATGTAGGAACAACCCCTGTCTC-3′. For siRNA transfections, RAW 264.7 cells were cultured in 12-well plates, and transfections were performed using FuGENE 6 (Roche Applied Science, Indianapolis, IN) in mixture with 2 µg of siRNA per well. After 72 hours, the cells were stimulated with 10^3^ bacteria per cell or with 10 µg of poly∶IC (InvivoGen, San Diego, CA). Accell SMARTpool siRNA duplexes targeting mouse STING (catalog n° E-055528), and Accell siRNA delivery media were purchased from Dharmacon (Thermo Fischer Scientific, Lafayette, CO). For STING siRNA-mediated knockdown, RAW 264.7 cells were first pretreated with the indicated siRNA or control duplexes for 72 hours in Accell siRNA media. Then the culture medium was replaced and the cells stimulated with 10^3^ bacteria/cell, DNA (1 µg/well) or poly I∶C (10 µg/mL; InvivoGen, San Diego, CA). Messenger RNA silencing was confirmed by quantitative SyBR-Green RT-PCR. Briefly, total RNA was extracted with TRIzol (GIBCO BRL) and further reverse-transcribed into cDNA with Ready-To-Go RT-PCR Beads (GE Healthcare, Buckinghamshire, UK) and real-time PCR was performed as described. For RNA polymerase III studies, Raw 264.7 cells were pretreated with the Pol-III inhibitor, ML-60218 (Calbiochem, Darmstadt, Germany), at 0.1 mM or 1.0 mM for 10 hours and then stimulated with 10^3^ bacteria/cell, 1 µg of purified *Brucella* DNA, or 10 µg/mL of poly I∶C (InvivoGen, San Diego, CA) for 3 hours. The supernatants were collected and the total RNA was extracted from the cells to access the expression of *IFN-β* by real-time RT-PCR as described above.

### Statistical analysis

The differences in IFN-β, IFN-γ, IL-17 and NO levels between wild type and IFN-αβR KO mice were analyzed by Student's *t* test using two-tail distribution. The differences in the relative expression of target genes were analyzed by Student's *t* test, using one tail distribution and heteroscedastic variance.

## Supporting Information

Figure S1 **Lack of type I IFN signaling is not related to IL-17A production in** ***Brucella*** **-infected splenocytes.** Spleen cells from *B. abortus* S2308 infected 129Sv/Ev or IFN-αβR KO mice after two-weeks of infection were cultured with 10^2^ bacteria/cell, ConA (5 µg/ml) or medium alone for 72 hrs and IL-17A was detected by ELISA. Error bars represent the mean ±SD. Similar results were obtained in two-independent experiments. Statistically significant difference from medium alone compared to bacteria or ConA is denoted by an asterisk (p<0.05).
(TIF)

Figure S2 ***Brucella*** **DNA but not its LPS or lipidated-Omp19 induces IFN-β expression in macrophages.** BMDM from C57BL/6, TLR2 or TLR4 KO mice were infected with *B. abortus* strain S2308 (10^3^ bact/cell), stimulated with *Brucella* DNA (1 µg/5×10^5^ cells), *Brucella* DNA treated with DNAse (0.2 U/µg DNA), poly I∶C (10 µg/ml), *Brucella* LPS (10 µg/ml), *Brucella* L-Omp19 (25 µg/ml) or medium alone. Twenty four hours after stimulation of the cells, total RNA was harvested and mRNA levels of *IFN-β* was determined by real-time RT-PCR and normalized to *β-actin*. Error bars represent the mean ±SD of samples assayed in triplicate. Similar results were obtained in two-independent experiments. Statistically significant difference of *IFN-β* levels from stimulated macrophages versus non-stimulated cells is denoted by an asterisk and from *Brucella* DNA compared to *Brucella* DNA treated with DNAse with two asterisks (p<0.05).
(TIF)

## References

[1] Franco MP, Mulder M, Gilman RH, Smits HL (2007). Human brucellosis.. Lancet Infect Dis.

[2] Boschiroli ML, Foulongne V, O'Callaghan D (2001). Brucellosis: a worldwide zoonosis.. Curr Opin Microbiol.

[3] Golding B, Scott DE, Scharf O, Huang YL, Zaitseva M (2001). Immunity and protection against *Brucella abortus*.. Microbes Infect.

[4] Murphy EA, Sathiyaseelan J, Parent MA, Zou B, Baldwin CL (2001). Interferon-gamma is crucial for surviving a *Brucella abortus* infection in both resistant C57BL/6 and susceptible BALB/c mice.. Immunology.

[5] Oliveira SC, Splitter GA (1995). CD8+ type 1 CD44hi CD45 RBlo T lymphocytes control intracellular *Brucella abortus* infection as demonstrated in major histocompatibility complex class I- and class II-deficient mice.. Eur J Immunol.

[6] Giambartolomei GH, Zwerdling A, Cassataro J, Bruno L, Fossati CA (2004). Lipoproteins, not lipopolysaccharide, are the key mediators of the proinflammatory response elicited by heat-killed *Brucella abortus*.. J Immunol.

[7] Campos MA, Rosinha GM, Almeida IC, Salgueiro XS, Jarvis BW (2004). Role of Toll-like receptor 4 in induction of cell-mediated immunity and resistance to *Brucella abortus* infection in mice.. Infect Immun.

[8] Huang LY, Ishii KJ, Akira S, Aliberti J, Golding B (2005). Th1-like cytokine induction by heat-killed *Brucella abortus* is dependent on triggering of TLR9.. J Immunol.

[9] Macedo GC, Magnani DM, Carvalho NB, Bruna-Romero O, Gazzinelli RT (2008). Central role of MyD88-dependent dendritic cell maturation and proinflammatory cytokine production to control *Brucella abortus* infection.. J Immunol.

[10] Oliveira SC, de Oliveira FS, Macedo GC, de Almeida LA, Carvalho NB (2008). The role of innate immune receptors in the control of *Brucella abortus* infection: toll-like receptors and beyond.. Microbes Infect.

[11] Isaacs A, Lindenmann J, Valentine RC (1957). Virus interference. II. Some properties of interferon.. Proc R Soc Lond B Biol Sci.

[12] Van den Broek MF, Müller U, Huang S, Aguet M, Zinkernagel RM (1995). Antiviral defense in mice lacking both alpha/beta and gamma interferon receptors.. J Virol.

[13] Monroe KM, McWhirter SM, Vance RE (2010). Induction of type I interferons by bacteria.. Cell Microbiol.

[14] O'Connell RM, Saha SK, Vaidya SA, Bruhn KW, Miranda GA (2004). Type I interferon production enhances susceptibility to *Listeria monocytogenes* infection.. J Exp Med.

[15] Auerbach V, Brockstedt DG, Meyer-Morse N, O'Riordan M, Portnoy DA (2004). Mice lacking the type I interferon receptor are resistant to *Listeria monocytogenes*.. J Exp Med.

[16] Manca C, Tsenova L, Bergtold A, Freeman S, Toney M (2001). Virulence of a *Mycobacterium tuberculosis* clinical isolate in mice is determined by failure to induce Th1 type immunity and is associated with induction of IFN-alpha/beta.. Proc Natl Acad Sci USA.

[17] Qiu H, Fan Y, Joyee AG, Wang S, Han X (2008). Type I IFNs enhance susceptibility to *Chlamydia muridarum* lung infection by enhancing apoptosis of local macrophages.. J Immunol.

[18] Schiavoni G, Mauri C, Carlei D, Belardelli F, Pastoris MC (2004). Type I IFN protects permissive macrophages from *Legionella pneumophila* infection through an IFN-γ-independent pathway.. J Immunol.

[19] Gold JA, Hoshino Y, Hoshino S, Jones MB, Nolan A (2004). Exogenous gamma and alpha/beta interferon rescues human macrophages from cell death induced by *Bacillus anthracis*.. Infect Immun.

[20] Mancuso G, Midiri A, Biondo C, Beninati C, Zummo S (2007). Type I IFN signaling is crucial for host resistance against different species of pathogenic bacteria.. J Immunol.

[21] Yoneyama M, Fujita T (2008). Structural mechanism of RNA recognition by the RIG-I-like receptors.. Immunity.

[22] Bieger CD, Nierlich DP (1989). Distribution of 5′-triphosphate termini on the mRNA of *Escherichia coli*.. J Bacteriol.

[23] Ablasser A, Bauernfeind F, Hartmann G, Latz E, Fitzgerald KA (2009). RIG-I-dependent sensing of poly(dA∶dT) through the induction of an RNA polymerase III-transcribed RNA intermediate.. Nat Immunol.

[24] Chiu YH, Macmillan JB, Chen ZJ (2009). RNA polymerase III detects cytosolic DNA and induces type I interferons through the RIG-I pathway.. Cell.

[25] Hornung V, Ellegast J, Kim S, Brzozka K, Jung A (2006). 5′-Triphosphate RNA is the ligand for RIG-I.. Science.

[26] Ishikawa H, Barber GN (2008). STING is an endoplasmic reticulum adaptor that facilitates innate immune signalling.. Nature.

[27] Prantner D, Darville T, Nagarajan UM (2010). Stimulator of IFN gene is critical for induction of IFN-beta during *Chlamydia muridarum* infection.. J Immunol.

[28] Ishii KJ, Akira S (2006). Innate immune recognition of, and regulation by, DNA.. Trends Immunol.

[29] Stetson DB, Medzhitov R (2006). Type I interferons in host defense.. Immunity.

[30] Al Moussawi K, Ghigo E, Kalinke U, Alexopoulou L, Mege JL (2010). Type I interferon induction is detrimental during infection with the Whipple's disease bacterium, *Tropheryma whipplei*.. PLoS Pathog.

[31] Roux CM, Rolan HG, Santos RL, Beremand PD, Thomas TL (2007). *Brucella* requires a functional Type IV secretion system to elicit innate immune responses in mice.. Cell Microbiol.

[32] den Hartigh AB, Rolán HG, de Jong MF, Tsolis RM (2008). VirB3 to VirB6 and VirB8 to VirB11, but not VirB7, are essential for mediating persistence of *Brucella* in the reticuloendothelial system.. J Bacteriol.

[33] Doyle S, Vaidya S, O'Connell R, Dadgostar H, Dempsey P (2002). IRF3 mediates a TLR3/TLR4-specific antiviral gene program.. Immunity.

[34] Charrel-Dennis M, Latz E, Halmen KA, Trieu-Cuot P, Fitzgerald KA (2008). TLR-independent type I interferon induction in response to an extracellular bacterial pathogen via intracellular recognition of its DNA.. Cell Host Microbe.

[35] Taniguchi T, Takaoka A (2002). The interferon-alpha/beta system in antiviral responses: a multimodal machinery of gene regulation by the IRF family of transcription factors.. Curr Opin Immunol.

[36] Carrero JA, Calderon B, Unanue ER (2004). Type I interferon sensitizes lymphocytes to apoptosis and reduces resistance to *Listeria* infection.. J Exp Med.

[37] Stanley SA, Johndrow JE, Manzanillo P, Cox JS (2007). The Type I IFN response to infection with *Mycobacterium tuberculosis* requires ESX-1-mediated secretion and contributes to pathogenesis.. J Immunol.

[38] Ordway D, Henao-Tamayo M, Harton M, Palanisamy G, Troudt J (2007). The hypervirulent *Mycobacterium tuberculosis* strain HN878 induces a potent TH1 response followed by rapid down-regulation.. J Immunol.

[39] Nagarajan UM, Prantner D, Sikes JD, Andrews CW, Goodwin AM (2008). Type I interferon signaling exacerbates *Chlamydia muridarum* genital infection in a murine model.. Infect Immun.

[40] Coers J, Vance RE, Fontana MF, Dietrich WF (2007). Restriction of *Legionella pneumophila* growth in macrophages requires the concerted action of cytokine and Naip5/Ipaf signalling pathways.. Cell Microbiol.

[41] Henry T, Brotcke A, Weiss DS, Thompson LJ, Monack DM (2007). Type I interferon signaling is required for activation of the inflammasome during *Francisella* infection.. J Exp Med.

[42] Rothlin CV, Ghosh S, Zuniga EI, Oldstone MB, Lemke G (2007). TAM receptors are pleiotropic inhibitors of the innate immune response.. Cell.

[43] Salcedo SP, Marchesini MI, Lelouard H, Fugier E, Jolly G (2008). *Brucella* control of dendritic cell maturation is dependent on the TIR-containing protein Btp1.. PLoS Pathog.

[44] Rayamajhi M, Humann J, Penheiter K, Andreasen K, Lenz LL (2010). Induction of IFN-alphabeta enables *Listeria monocytogenes* to suppress macrophage activation by IFN-gamma.. J Exp Med.

[45] Baldwin CL, Goenka R (2006). Host immune responses to the intracellular bacteria *Brucella*: does the bacteria instruct the host to facilitate chronic infection?. Crit Rev Immunol.

[46] Carrero JA, Calderon B, Unanue ER (2004). Type I interferon sensitizes lymphocytes to apoptosis and reduces resistance to *Listeria* infection.. J Exp Med.

[47] Guo B, Chang EY, Cheng G (2008). The type I IFN induction pathway constrains Th17-mediated autoimmune inflammation in mice.. J Clin Invest.

[48] Hamada S, Umemura M, Shiono T, Tanaka K, Yahagi A (2008). IL-17A produced by gammadelta T cells plays a critical role in innate immunity against *Listeria monocytogenes* infection in the liver.. J Immunol.

[49] Henry T, Kirimanjeswara GS, Ruby T, Jones JW, Peng K (2010). Type I IFN signaling constrains IL-17A/F secretion by {gamma}{delta} T cells during bacterial infections.. J Immunol.

[50] Fitzgerald KA, McWhirter SM, Faia KL, Rowe DC, Latz E (2003). IKKepsilon and TBK1 are essential components of the IRF3 signaling pathway.. Nat Immunol.

[51] Gratz N, Siller M, Schaljo B, Pirzada ZA, Gattermeier I (2008). Group A streptococcus activates type I interferon production and MyD88-dependent signaling without involvement of TLR2, TLR4, and TLR9.. J Biol Chem.

[52] Glomski IJ, Fritz JH, Keppler SJ, Balloy V, Chignard M (2007). Murine splenocytes produce inflammatory cytokines in a MyD88-dependent response to *Bacillus anthracis* spores.. Cell Microbiol.

[53] Lapaque N, Muller A, Alexopoulou L, Howard JC, Gorvel JP (2009). *Brucella abortus* induces Irgm3 and Irga6 expression via type-I IFN by a MyD88-dependent pathway, without the requirement of TLR2, TLR4, TLR5 and TLR9.. Microb Pathog.

[54] Kagan JC, Su T, Horng T, Chow A, Akira S (2008). TRAM couples endocytosis of Toll-like receptor 4 to the induction of interferon-beta.. Nat Immunol.

[55] Pizarro-Cerda J, Moreno E, Sanguedolce V, Mege JL, Gorvel JP (1998). Virulent *Brucella abortus* prevents lysosome fusion and is distributed within autophagosome-like compartments.. Infect Immun.

[56] Pizarro-Cerda J, Meresse S, Parton RG, van der Goot G, Sola-Landa A (1998). *Brucella abortus* transits through the autophagic pathway and replicates in the endoplasmic reticulum of nonprofessional phagocytes.. Infect Immun.

[57] Trant CG, Lacerda TL, Carvalho NB, Azevedo V, Rosinha GM (2010). The *Brucella abortus* phosphoglycerate kinase mutant is highlyattenuated and induces protection superior to that of vaccine strain 19 inimmunocompromised and immunocompetent mice.. Infect Immun.

[58] Wilson K, Ausubel FM, Brent R, Kimgston RE, Moore DD, Seidman JG, Smith JA, Struhl K (1990). Preparation of genomic DNA from bacteria.. Current protocols in molecular biology.

